# A Faraday laser lasing on Rb 1529 nm transition

**DOI:** 10.1038/s41598-017-09501-w

**Published:** 2017-08-21

**Authors:** Pengyuan Chang, Huanfa Peng, Shengnan Zhang, Zhangyuan Chen, Bin Luo, Jingbiao Chen, Hong Guo

**Affiliations:** 1 State Key Laboratory of Advanced Optical Communication System and Network, Institute of Quantum Electronics, School of Electronics Engineering and Computer Science, Peking University, Beijing, 100871 China; 2 State Key Laboratory of Information Photonics and Optical Communications, Beijing University of Posts and Telecommunications, Beijing, 100876 China

## Abstract

We present the design and performance characterization of a Faraday laser directly lasing on the Rb 1529 nm transition (Rb, 5*P*_3/2_ − 4*D*_5/2_) with high stability, narrow spectral linewidth and low cost. This system does not need an additional frequency-stabilized pump laser as a prerequisite to preparing Rb atom from 5S to 5P excited state. Just by using a performance-improved electrodeless discharge lamp-based excited-state Faraday anomalous dispersion optical filter (LESFADOF), we realized a heterogeneously Faraday laser with the frequency corresponding to atomic transition, working stably over a range of laser diode (LD) current from 85 mA to 171 mA and the LD temperature from 11 °C to 32 °C, as well as the 24-hour long-term frequency fluctuation range of no more than 600 MHz. Both the laser linewidth and relative intensity noisy (RIN) are measured. The Faraday laser lasing on Rb 1529 nm transition (telecom C-band) can be applied to further research on metrology, microwave photonics and optical communication systems. Besides, since the transitions correspongding to the populated excited-states of alkali atoms within lamp are extraordinarily rich, this scheme can increase the flexibility for choosing proper wavelengths for Faraday laser and greatly expand the coverage of wavelength corresponding to atomic transmission for laser frequency stabilization.

## Introduction

The semiconductor lasers today have the advantages of small size, light weight, longevity, energy-efficiency, tunability^1^. However, the free-running semiconductor lasers without feedback have several negative features, including frequency long-term drift, wideband, frequency susceptibility to laser diode (LD) temperature and driving current, etc^2^. In most instances, semiconductor lasers were used on optical pumping and low resolution spectroscopy in the past. For example, although semiconductor lasers were recognized as suitable light sources for gas-absorption spectroscopy due to their reliability, compact size, and are applied to gas sensors nowadays^3^, the linewidth of semiconductor lasers is typically in the MHz range which is too wide to meet the requirement of fiber-optic sensing^4^. One of the main drawbacks of semiconductor lasers is that, it is difficult to obtain narrowband and easily tunable output. With the development of new wavelength control techniques, the situation has been improved considerably. Various techniques have been used to control and narrow the spectral output of semiconductor lasers, such as interference filters^5^, gratings^6, 7^, Fabry–Perot etalons^8, 9^ and Pound–Drever–Hall technology^10–13^. Furthermore, it is well known that laser frequency can also be stabilized by using methods of atomic or molecular absorption line^14^, including frequency-modulation spectroscopy^15^, polarization spectroscopy^16^, modulation transfer spectrum^17^, etc.

Since Faraday anomalous dispersion optical filter (FADOF) tecnology was introduced in 1956^18^, the application of FADOF to the organic dye lasers mode selection has been carried out^19, 20^. The output frequency of Faraday laser^21–25^ is stabilized on the atomic resonance absorption line by adjusting magnitude and temperature of FADOF to optimal values, while absolutely immune to the changes of LD temperature and driving current^21^ in a large scale. Hence, the performance of the Faraday laser is similar to He-Ne laser when it is powered on, while the output frequency of the Faraday laser exactly correspondes to the Doppler resonance absorption line of the atoms^22^. The Faraday laser can be applied to a number of diverse applications, such as atomic clock^26^, atomic interferometer, atomic gyroscope, magnetometer^27, 28^ and free-space communications^29^. With the increasing demand of various fields of FADOF^30, 31^, the development of FADOF technology is already in a fairly mature stage. In recent years, various bands of FADOFs have been developed, including 423 nm (Ca)^32, 33^, 455 nm (Cs)^34, 35^, 461 nm (Sr)^36^, 589 nm (Na)^37^, 780 nm (Rb)^38–42^, 795 nm (Rb)^43^, 852 nm (Cs)^44^, 1529 nm (Rb)^45, 46^, etc^47–49^.

Recently, a series of experiments on Faraday lasers have been conducted. Although the Faraday laser lasing on 780 nm ground-state transition was reported^21, 22^, the excited-state Faraday laser has never been realized due to the low transmittance^46^ of the electrodeless discharge lamp-based Faraday anomalous dispersion optical filter (LESFADOF) and the indispensability of an additional frequency-stabilized pump laser^50–53^. In conventional 1529 nm optical wavelength standards^50^, the method of utilizing an optically pumped Rb vapor cell which exhibits resonances at 1529 nm makes the whole system costly, bulky and relatively high in complexity. As Rb 1529 nm excited-state transition line from 5P to 4D has been widely accepted as wavelength standard in metrology, microwave photonics and optical fiber telecommunication^52^. Recently, much effort is devoted toward the realization of practical frequency standard in the telecom C-band (1528 nm–1563 nm) on application of optical communication systems^29^.

In this paper, we successfully demonstrated a Faraday laser directly lasing on the Rb 1529 nm transition from $5{P}_{3/2}-4{D}_{5/2}$ state by utilizing a performance-improved LESFADOF, instead of a frequency-locked pump laser. The output frequency of Faraday laser is insensitive to the changes of LD temperature and driving current. The measured results indicate that the Faraday laser with the frequency corresponding to atomic transitions works stably over a wide range of the LD current from 85 mA to 171 mA and the temperature from 11 °C to 32 °C. Both the laser linewidth and relative intensity noisy (RIN) are measured. The successful development of the 1529 nm Faraday laser provides an innovative method for laser frequency stabilization. The new technology of LESFADOF-based Faraday laser greatly expands the applications of excited-state transitions of alkali atoms, and provides the flexibility for tuning the laser frequency to a desired wavelength corresponding to atomic transition. In addition, the low-cost Faraday laser lasing on Rb 1529 nm transition in telecommunication wavelength window can be applied to metrology^54^, microwave photonics^55^ and optical fiber telecommunication^29^ and other practical applications^56^.

## Results

### The performance of LESFADOF at Rb 1529 nm transition

**Figure 1:**
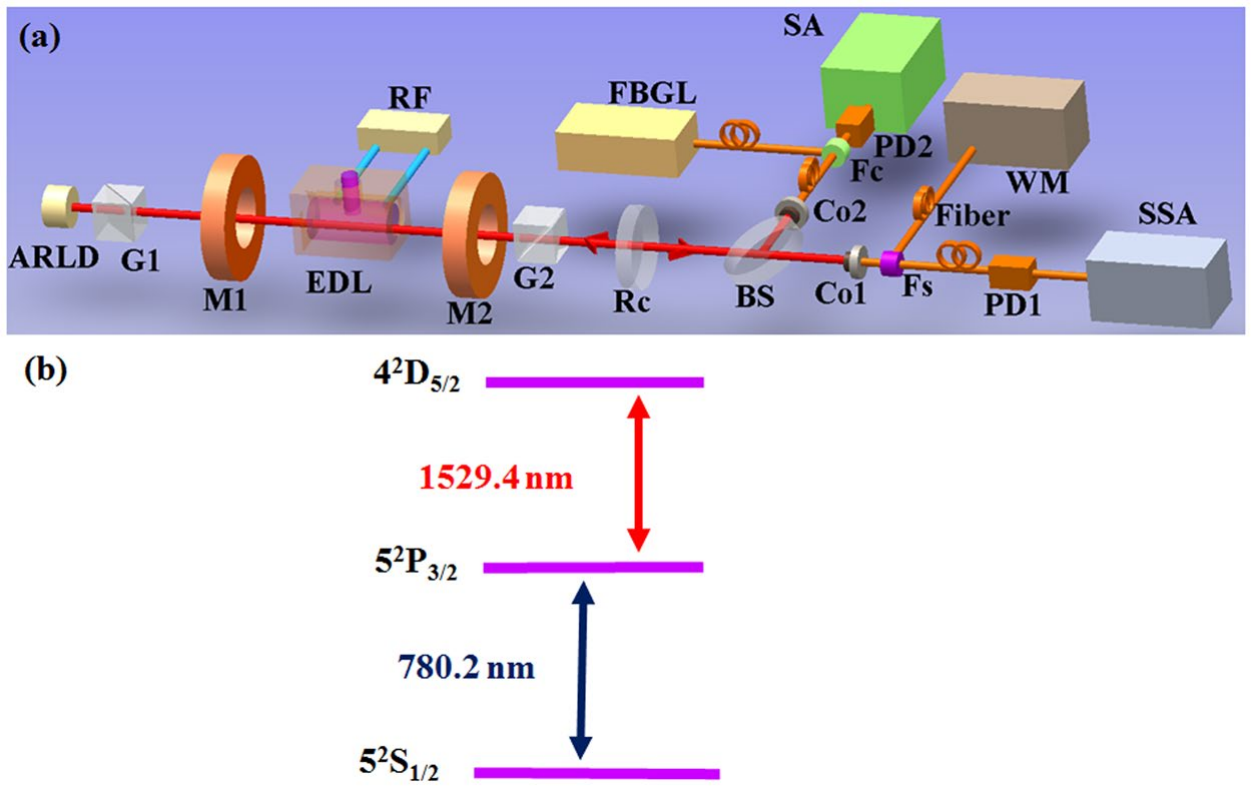
Schematic of the LESFADOF-based Faraday laser lasing on Rb 1529 nm transition with the testing system and relevant energy level diagram. (**a**) Experimental setup of the LESFADOF-based Faraday laser lasing on Rb 1529 nm transition and the measurement system. ARLD, antireflection-coated laser diode; RF, radio-frequency module; M1 and M2: a pair of permanent magnets; EDL, electrodeless discharge lamp; G1 and G2, a pair of Glan–Taylor prisms; Rc, cavity mirror with reflectivity of 80% and the transmission of 20% for 1529 nm; BS, 50% beam splitter for 1529 nm; Co1 and Co2, optical fiber coupler for 1529 nm; Fc, fiber combiner; Fs, fiber splitter; Fiber, single-mode fiber; PD1 and PD2, photodiode; FBGL, fiber Bragg grating laser; SSA, signal source analyzer; WM, wavelength meter; SA, signal analyzer. (**b**) Relevant energy level diagram.

**Figure 2:**
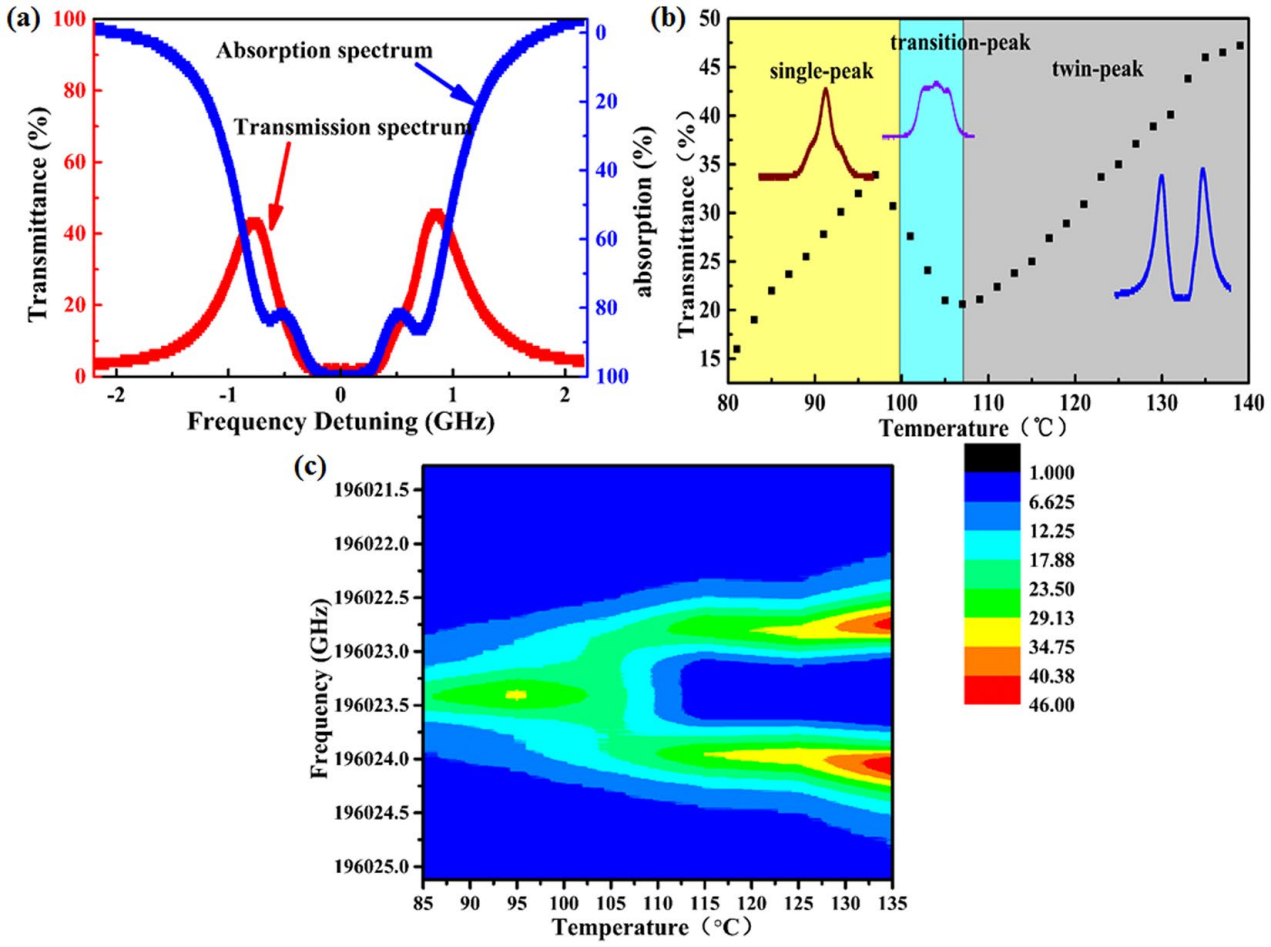
**(a**) The measured absorption spectrum (red curve) and measured transmission spectrum (blue curve) of the improved LESFADOF. The measured absorption spectrum refers to the absorption of the Rb vapor cell, the transmission spectrum refers to the transmission of the entire LESFADOF. **(b)** The peak transmittance of the LESFADOF at Rb 1529 nm transition as a function of the cell temperature with a static axial magnetic field of 500 G across the Rb vapour cell. The distributing region with different color shows the different transmission spectrum line-shape. **(c)** The transmittance of the whole LESFADOF as a function of the Rb cell temperature from 85 °C to 135 °C with a static axial magnetic field of 500 G across the Rb vapour cell.

The experimental setup of the Faraday laser system is depicted in Fig. 1(a). In order to measure the performance of the LESFADOF, we conducted an experiment which is descripted in section of methods. Figure 2(a) shows the transmission spectrum of the entire LESFADOF (red curve) and absorption spectrum of the Rb vapor cell (blue curve) at Rb 1529 nm transition. The peak transmittance varies following the changes of the Rb cell temperature, as shown in Fig. 2(b), the black dotted line shows the transmittance as a function of the Rb cell temperature. The distributing region with different color shows different transmission spectrum line-shape of single-peak, transition-peak, twin-peak, respectively. Figure 2(c) shows the transmittance of the whole LESFADOF as a function of the Rb cell temperature from 85 °C to 135 °C with a static axial magnetic field of 500 G across the Rb vapour cell. By adjusting the Rb cell temperature, we have found an optimal working point for a maximized transmittance. Under the condition of the magnetic field of 500 G and the Rb cell temperature of 135 °C, the twin-peak line-shaped transmission spectrum corresponding to Rb $5{P}_{3/2}-4{D}_{5/2}$ transition (1529.4 nm in vacuum) with a maximum transmittance of 46% is obtained (without taking into account the system loss and fluorescence background). Compared with the maximum transmisstance of the entire LESFADOF in the ref. 45, it is improved from 21.9%^46^ to 46%, which plays a key role in this work.

### Relative intensity noise

**Figure 3:**
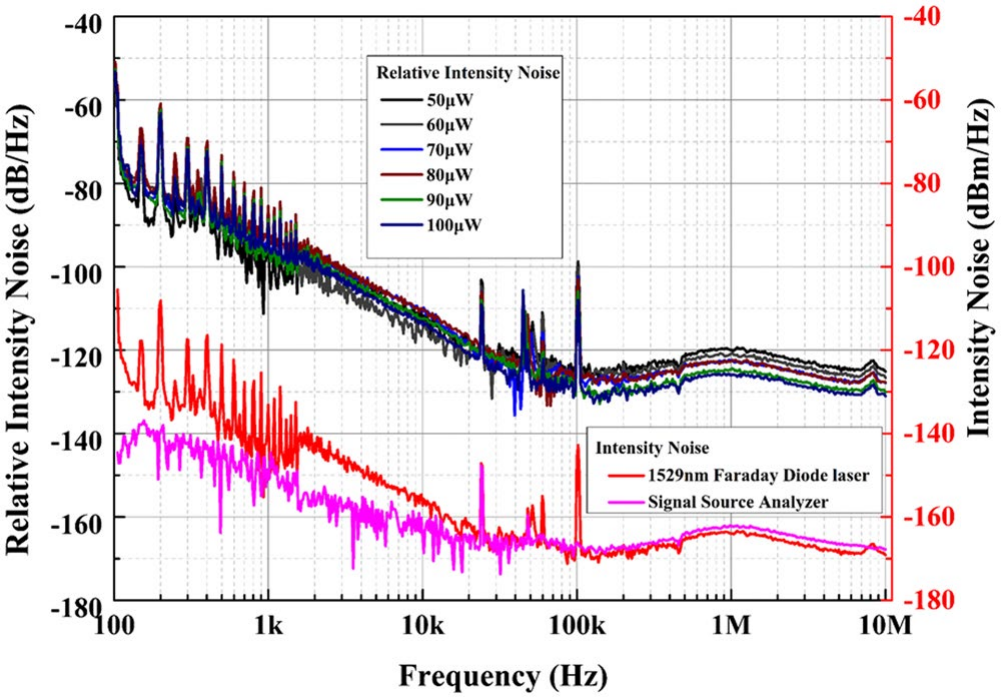
The two bottom colored lines refer to the electrical-domain intensity noise of the Faraday laser lasing on Rb 1529 nm transition (red curve) and signal source analyzer (pink curve). The six upper colored lines refer to measured relative intensity noise of the Faraday laser with different optical power.

The optical power intensity fluctuations of the LD are characterized by relative intensity noise (RIN)^57^. To measure the RIN of the Faraday laser, we constructed a simple experiment setup, as depicted in Fig. 1(a). The optical signal is converted to electric signal by a photodiode, and the intensity noise of the electric signal is measured by signal source analyzer (SSA, Keysight E5052B). The noise measurement results are plotted in Fig. 3, with the tested frequency range from 100 Hz to 10 MHz. The intensity noise floor of the SSA is obtained without the input of the electric signal, as shown by the bottom pink curve in Fig. 3. With the electric signal transmitted to the SSA, the measured intensity noise is shown by bottom red curve. The intensity noise of the light at offset frequency below 30 kHz is higher than the intensity noise floor of the SSA. Nevertheless, the intensity noise of the optical signal can not be precisely measured at offset frequency above 30 kHz due to the limitation of the SSA sensitivity. By improving the power of the optical signal, the intensity noise above 30 kHz offset can be measured. The RIN measurement results with different optical power are shown by the six upper colored lines in Fig. 3, the results for different optical power are almost unchanged below 30 kHz offset. The RIN performance of the 1529 nm Faraday laser is measured to be −115 dB/Hz at 10 kHz offset when the optical power is 70 *μ*W. The optical power of the Faraday laser varies with the change of the Faraday laser driving current, and the measured colored line corresponds to fiber-coupled optical power of 50 *μ*W, 60 *μ*W, 70 *μ*W, 80 *μ*W, 90 *μ*W, 100 *μ*W, respectively.

### Laser linewidth

**Figure 4:**
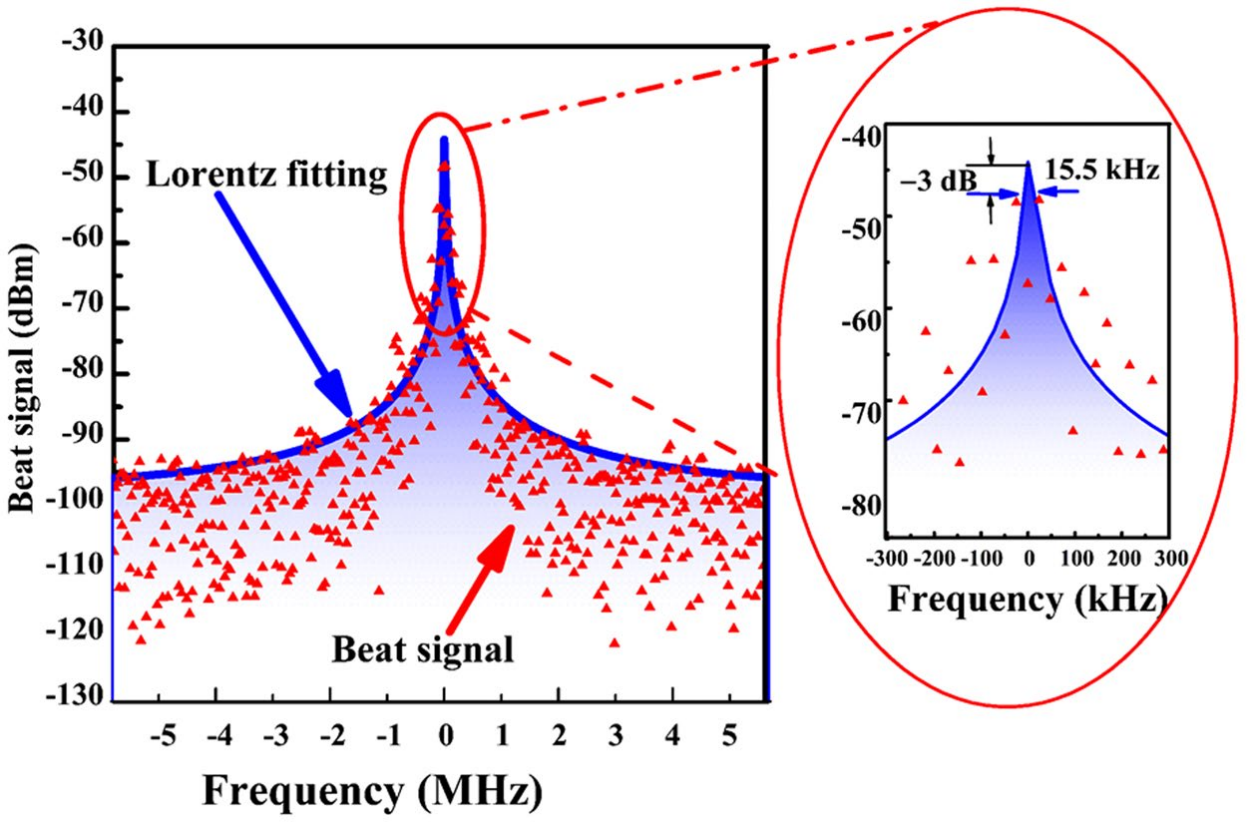
The spectrum of beat note between the Faraday laser (red triangles) and a FBGL with 10 kHz RBW and the Lorentzian fit curve (blue curve). The sweep time of the spectrum analyzer is 5 ms, and the video bandwidth is 10 kHz. The −3 dB linewidth of the Lorentzian fit curve is 15.5 kHz.

The measurement of the optical linewidth of the Faraday laser is implemented by using a heterodyne setup, as depicted in Fig. 1(a). The light beam of the Faraday laser is sent to optical fiber and combined with the light of a commercial tunable dual-mode Fiber Bragg Grating laser (FBGL) (PS-TNL) for beating on a photodiode. To measure the laser linewidth, the beat note between two lasers is sent to a signal analyzer (N9030A PXA, Agilent, US). The linewidth is measured by using a Lorentzian fit, and the result of beat-note is presented in Fig. 4, the −3 dB linewidth of the Lorentz fitting curve is 15.5 kHz. Since the FBGL has a linewidth of 1 kHz, the Faraday laser hence has a linewidth of 15.5 kHz.

### Insensitivity of the frequency of the Rb 1529 nm Faraday laser to the changes of LD temperature and current

**Figure 5:**
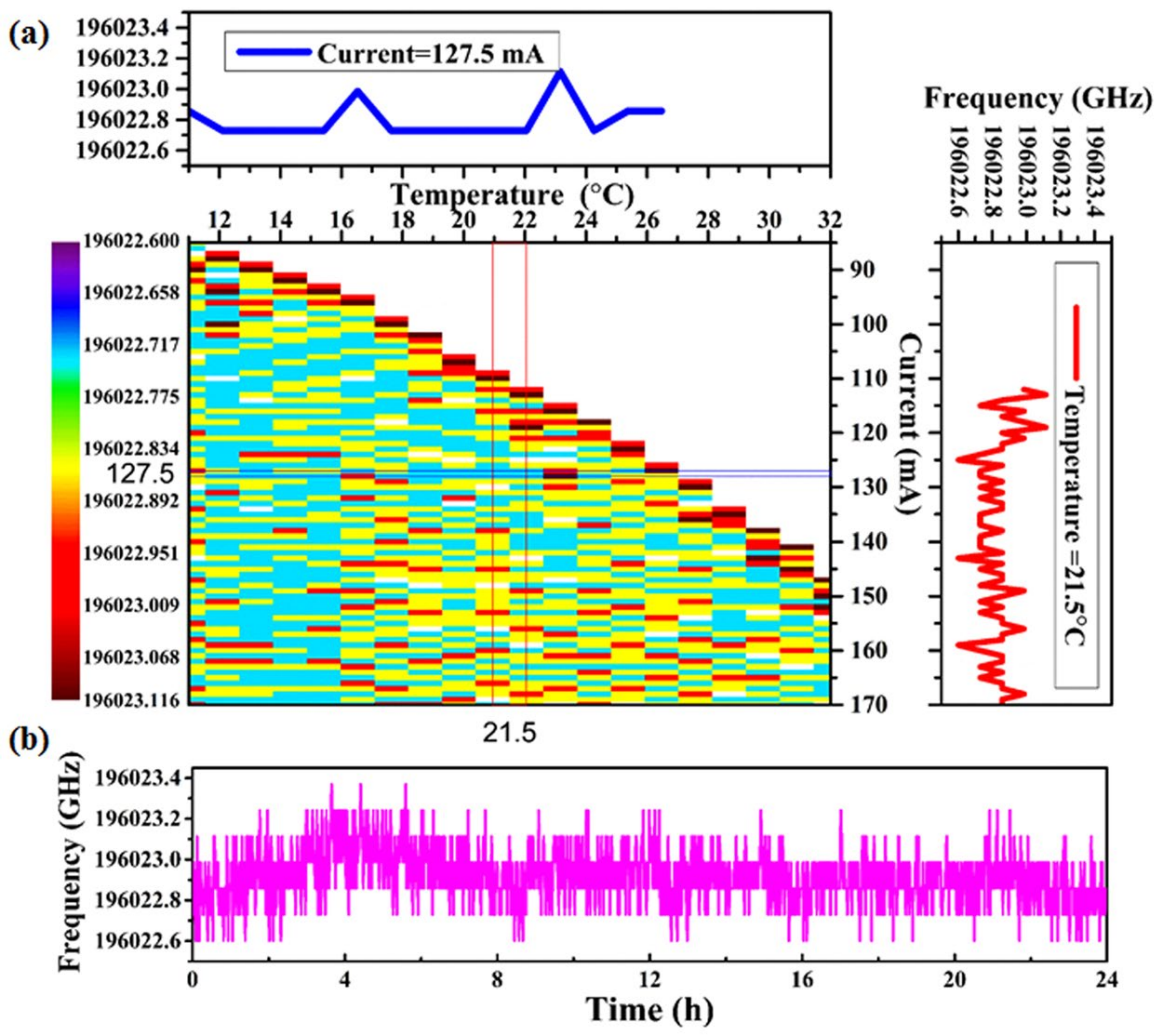
**(a)** The frequency of the Faraday laser as a function of the LD temperature and LD current, the LD temperature range from 11 °C to 32 °C and LD current range from 85 mA to 171 mA, the measured frequency values center on 196022.875 GHz, the threshold of Faraday laser increase along with the increase of LD temperature. The upper subfigure shows the frequency of the Faraday laser as a function of the LD temperature with fixed LD current of 127.5 mA (up blue curve). The right subfigure shows the frequency of the Faraday laser as a function of the LD current with fixed LD temperature of 21.5 °C (right red curve). **(b)** The 24-hour long-term frequency fluctuations of the Faraday laser with LD current of 141.5 mA and LD temperature of 21.5 °C, the Rb cell temperature is kept at 135 °C.

The astonishing advantage of the Faraday laser is that the exact operating frequency of Faraday laser is absolutely determined by Rb 1529 nm transition and insensitive to the changes of LD temperature and current. The frequency measurement results by using a wavelength meter (WM, ADVANTEST TQ8325) of our experiment are shown in Fig. 5(a). Under the condition of the LD temperature ranging from 11 °C to 32 °C and the current from 85 mA to 171 mA, the measured frequency of the Faraday laser centers on 196022.875 GHz corresponding to one of twin-peak line-shaped transmission spectrum with maximum transmittance of 46%. In the upper subfigure, the blue curve presents the frequency of the Faraday laser as a function of the LD temperature ranging from 11 °C to 27 °C with fixed LD current of 127.5 mA. In the right subfigure, the red curve shows the frequency of the Faraday laser as a function of the LD current ranging from 110 mA to 171 mA with fixed LD temperature of 21.5 °C, the maximum current is limited by LD power supply.

As shown in Fig. 2(b) and (c), when the Rb cell temperature is 135 °C, the transmission spectrum of the entire LESFADOF with maximum transmittance corresponds to laser frequency of 196022.875 GHz. That’s the reason why the laser frequency centers on 196022.875 GHz. The frequency fluctuation range of below 600 MHz primarily reasults from the mode instability of the optical cavity due to mechanical vibration and air flow. The Faraday laser we demonstrated has an optical cavity length of 60 cm, which provides optical feedback and has wide free spectrum range of 250 MHz. On the basis of previous studies^23, 24^, a longer fiber can be used as an extended cavity to limit the laser frequency to the center of transmission spectrum and reduce laser frequency long-term drift, which can further improve the frequency stability and narrow optical linewidth. Hence a fiber-based Faraday laser lasing on Rb 1529 nm with an ultra-narrow bandwidth can be realized.

The measured result of 24-hour long-term frequency fluctuations of the Faraday laser is shown in Fig. 5(b). Under the condition of LD current of 141.5 mA and LD temperature of 21.5 °C, the frequency fluctuation range is about 600 MHz within 24 hours. The Rb cell temperature remains at 135 °C in this measurement.

Besides, the laser frequency has been always stabilized at Rb 1529 nm excited-state transition when it is powered on more than 50 times during six months from beginning to the end of our experiment. Consequently, the reproducibility of the Faraday laesr is validated to be superior.

Furthermore, we measured the frequency of Faraday laser as a function of the Rb cell temperature (not depicted in this paper), the frequency fluctuation is about 500 MHz with the Rb cell temperature ranging from 128 °C to 178 °C, hence we can conclude that the frequency drift is only 40 kHz if Rb cell temperature control accuracy is 0.01 °C. It is proved that the cell temperature has little influence on the laser frequency, while we have not found that RF power has any effect on laser frequency yet.

## Discussion

In conclusion, we successfully demonstrated a proof-of-principle Faraday laser lasing on Rb 1529 nm transition by using a performance-improved LESFADOF compared with the previous LESFADOF in ref. 45. The frequency of the Faraday laser is insensitive to the changes of LD temperature and driving current while exactly resonant with the Rb 1529 nm transition. The Faraday laser wavelength in the telecommunication wavelength window potentially allows further applications on optical communication systems. Besides, since the excited-state transitions of alkali atoms are extraordinarily rich, this scheme can increase the flexibility for choosing proper wavelength for Faraday laser and greatly expand coverage of wavelength corresponding to atomic transition for laser frequency stabilization. Considering there are various FADOFs of different wavelengths with high transmittance, such as 461 nm FADOF which was studied by a hollow cathode lamp based FADOF (HCL-FADOF)^36^. The successful development of the LESFADOF-based Faraday laser predicts potential possibility for the implementation of Faraday laser based on HCL-FADOF^36^. Therefore, this method can greatly expand the application of other wavelengths corresponding to atomic transitions of about 70 kinds of elements, including hige melting point metals^36^. Furthermore, the successful implementation of the Faraday laser provides an innovative method for laser frequency stabilization, and the low-cost Faraday laser can be applied to metrology, microwave photonics and optical fiber telecommunication and other practical applications.

In addition, since our experiment is built on the optical bread board, both the mechanical vibration and air flow limit the stability of laser frequency. We can suppress intensity noise and frequency drift by approach of vibration isolation. Furthermore, lasers integrated with reflection-coated fiber as extended optical cavity have the potential for substantial reduction of noise. Hence a fiber-based Faraday laser can be used as a stable laser source for practical applications.

The total single path optical loss of the whole system is about 40%, mainly arising from optical loss of the cell windows, which is measured to be 35%. Optical loss impairs the transmitted power and hence increases the threshold of the Faraday laser system. The optical loss of the cell windows can be reduced by improving the window flatness and optical coating of the end windows.

In order to improve the practicability of the Faraday laser, we have been working on ways to optimize the working conditions of the Faraday laser, such as reducing the RF driving power and the temperature of the electrodeless discharge lamp (EDL), increasing the output power of the Faraday laser. In the experiment, we measured the performance of the laser beam emitted from cavity mirror (Rc) for coonvenience of measurement. In addition, a laser beam with power of 1.3 mW exits from G2, and the direction of the light is perpendicular to the horizontal plane, not depicted in Fig. 1(a). The polarization of the light at the output of G2 and Rc is orthogonal. In practical applications, we can also apply the laser beam which exiting G2 to practical applications.

## Methods

### Excited-state 1529 nm FADOF based on electrodeless discharge lamp

The LESFADOF is the key element of the frequency-fixed Faraday laser system. In this experiment, we utilized a home-made performance-improved electrodeless discharge lamp (EDL) and realized a maximum transmittance of 46% higher than the original transmittance of 21.9% in ref. 45. Atoms can be excited from $5{S}_{1/2}-5{P}_{3/2}$ by amplified radio-frequency rather than a frequency-locked pumping laser.

The physics of LESFADOF can be described as follows^45, 46^. Electrons and ions in the vapor cell are accelerated when the RF power is turned on, due to the collision of the buffer gas, more electrons and ions are generated simultaneously. Therefore, the Rb atoms are pumped from ground state to excited state by high-energy-state buffer gas atoms. Since the Rb EDL has rich spectra, among which 780 nm (corresponding to the transition of $5{S}_{1/2}-5{P}_{3/2}$) is one of strongest transition peak. The Rb EDL can be utilized to realize an excited-state 1529 nm FADOF. The relevant energy level diagram^58, 59^ is shown in Fig. 1(b).

The principle of how the LESFADOF works is very similar to a conventional FADOF^18^. The main difference is that an EDL instead of a vapor cell is used in this work. The experiment setup is shown in Fig. 1(a), briefly, the LESFADOF includes a home-made EDL, a pair of permanent magnets, and a pair of crossed Glan-Taylor prisms. To characterize the filter, the probe light is provided by a 1529 nm grating laser with the laser frequency scanned across the $5{P}_{3/2}-4{D}_{5/2}$ transition (1529.4 nm in vacuum; see Fig. 1(b)) by using a piezoelectric actuator, a photodetector is used to detect transmission spectrum of the entire LESFAOF and absorption spectrum of the vapor cell.

The EDL contains a Rb atom vapor cell filled with natural Rb and 2 torr Xe buffer gas with length of 15 cm and diameter of 2 cm. The radio-frequency (RF) driving power can be adjusted from 0 W to 60 W, the cell temperature is controlled by heating supply current. When the cell temperature is heated to 85 °C, the optimal operation condition of the LESFADOF is mainly determined by the RF driving power. When the RF driving power is higher than 15 W, the EDL is lighted up, while the Faraday laser can work stably. Hence, the low RF power of 15 W is sufficient for our system. Furthermore, the laser Frequency is not influenced by the changes of RF power. The permanent magnets create a static axial magnetic field of 500 G across the Rb vapour cell with the inhomogeneity of the magnetic field of less than 7%, which can be ignored, the magnetic field is measured in absence of the EDL and there wasn’t magnetic shielding.

### Experiment setup of the Faraday laser lasing on 1529 nm and measurement system

The experimental setup is schematically shown in Fig. 1(a). The Faraday laser with the length of optical cavity of 60 cm is composed by the LESFADOF, ARLD and Rc. The LESFADOF is the key element of the Faraday laser (See detailed description in last paragraph). The light emitted from the ARLD (Toptica LD-1550-0050-AR-2) is filtered by the LESFADOF and fed back into the ARLD by Rc. The light emitted from Rc is coupled into the single-mode optical fiber by using a coupler for measureing the frequency of the Faraday laser. The laser frequency is measured by a WM. The Laser linewidth is measured by beating between the Faraday laser and the FBGL on a photodiode. The light from each laser is sent to optical fiber for beating and the beat note is sent to a SA. The RIN of the Faraday laser is measured using a SSA, the influence of the setup noise has been minimized using SSA noise cancellation (10000 correlations). The optical power at the output of Rc ranges from 50 *μ*W to 700 *μ*W (free space) or from 15 *μ*W to 240 *μ*W (fiber coupled), the optical power can be further improved since the rated output power of the LD are not reached.
